# Psychosocial and Clinical Profiles of the Cases Visiting the Emergency Department Due to Accidental Self-harm and Suicide Attempts in Doha, Qatar: A Retrospective Study

**DOI:** 10.1007/s10597-020-00650-3

**Published:** 2020-06-06

**Authors:** Hassen Al-Amin, Rajvir Singh, Mohamad Abdulrazzak, Suhaila Ghuloum

**Affiliations:** 1 Department of Psychiatry, Weill Cornell Medicine, Doha, Qatar; 2 Department of Cardiology, Hamad Medical Corporation, Doha, Qatar; 3 Department of Laboratory Medicine and Pathology, Hamad Medical Corporation, Doha, Qatar; 4 Department of Psychiatry, Hamad Medical Corporation, P.O. Box 3050, Doha, Qatar; 5 Weill Cornel Medicine-Qatar, Doha, Qatar

**Keywords:** Suicide attempt, Accidental self-harm, Parasuicide, Emergency department, Qatar

## Abstract

The aims of this study were to retrospectively assess the profiles of subjects with suicide attempts and self-harm in Doha, Qatar; and whether the available data were complete. We reviewed all the records of fatal and non-fatal suicides together with accidental self-ham cases seen in the major Emergency Department in Doha, over a one-year period. There was 37 completed suicide, mostly male expatriates in mid 30 s who died by hanging. In cases with suicide intent (N = 270), more males were admitted to Psychiatry than women. Overdose was the common method and the majority had mood disorders. In self-harm cases with no suicide intent (N = 150) the majority were not seen by Psychiatry. The profiles of suicide cases in Qatar are similar to those reported internationally. However, there is a major need to establish a comprehensive system to register and assess all self-harm patients in Qatar.

## Introduction

World Health Organization (WHO) defines suicide as “the act of deliberately killing oneself” and self-harm as “an act with non-fatal outcome, in which an individual deliberately initiates a non-habitual behavior…” (WHO 2014). Many acts of self-harm are not associated with suicidal intent. They may be an attempt to communicate with others, to call for help from others or a way of obtaining relief from a problematic and otherwise overwhelming situation or emotional state (Hjelmeland et al. 2002). WHO recognizes suicide as a global public health issue, reporting that 800,000 people die from suicide each year (WHO 2014). It has also been shown that for every suicide death there are 20–50 suicide attempts. In people between the 15 and 29 years old, suicide is the first cause of death in several countries and the second cause in many other countries. Worldwide, 78% of suicides occur in low- to middle-income countries (WHO Suicide 2018). Following an act of self-harm, the rate of suicide increases to 50–100 times the rate in the general population (Hawton et al. 2003; Owens et al. 2002). Men who self-harm are more than twice as likely to die by suicide as women and the risk increases significantly with age for both genders (Hawton et al. 2003). Studies of the method of suicide suggested that people who survived a medically serious suicide attempt might have a poorer outcome concerning life expectancy (Beautrais 2003). Characteristics of the attempt, such as high lethality and careful planning, are thought to suggest a higher possibility of a future fatal suicide (Runeson et al. 2010). Thus, reduction in suicidal behavior is among the WHO’s Health for all targets (WHO 2014).

The above determinants and risk factors for suicide are also affected by culture and ethnicity (Choo et al. 2017). International suicide rates are known to be higher for males, but there are significant differences in this male to female ratio among different countries. In developing countries, these rates are much higher than those in low- to middle-income countries (WHO 2014). Furthermore, compared to other countries, suicide rates are lowest in the Eastern Mediterranean Region (EMR). Several explanations have been given to this including the fact that many of the EMR countries do not have a suicide or self-harm registry system, which would result in under-reporting (Sarfraz and Castle 2002). Suicide is condemned in Islam, as in most world religions. With Islam being the most common faith in the EMR, and the laws in the region largely religion-based, a suicidal act is considered a taboo. Thus, individuals who have attempted suicide might avoid going to the hospital for treatment, as the attempt will be reported to the police (Khan 2007; Lester 2006). In addition, the stigmatization of suicide and certain non-acceptance to the religious groups after a suicidal attempt result in its misunderstanding, non-identification and inevitably underreporting (Sarfraz and Castle 2002; Pritchard and Amanullah 2007).

Most of the international studies in the developed countries rely on national suicide registries to collect data, measure progress, and recommend changes in policies and procedures accordingly. A recent study using the national database from USA (Canner et al. 2018) reported a stable annual incidence rate of ED visits for suicide attempts of 163–173 per 100,000 between 2006 and 2013. Poisoning was the most common method used. More visits were by females, but more males used violent methods. The mean age was in the early 30 s with a peak for young individuals between 15 and 19 years. The majority (over 80%) had a concurrent mental illness, mostly mood disorders. A meta-analysis from Japan reported the same profile, but the prevalence rate of suicide attempts in ED was up to 4.7% among all ED visits (Kawashima et al. 2014). In this regard, most of the published literature from the EMR, including the Middle East North Africa (MENA) is outdated (Morovatdar et al. 2013). A community-based study in Iran revealed the lifetime prevalence of suicidal attempts to be 3.3% (Malakouti et al. 2009). A follow up study in Iran interviewed adolescent patients admitted with suicidal attempts, finding the sociocultural context of suicide (such as failure in love, family problems, pressure of high expectations, and poverty) to be significant (Keyvanara and Haghshenas 2011). The absence of data might also skew results as suggested in a study of six cities in Pakistan where with the variations reported on suicide rates (from 0.4 to 2.9 per 100,000 population), the authors considered the rates to be underestimated (Khan et al. 2008). A recent study carried out in Dubai found that suicide rates were seven times higher among the expatriate community than the nationals where 78% of the suicide cases were Indian. Suicide rates were higher amongst single, expatriate, and employed males (Dervic et al. 2012).

Still, there is little existent community studies or registry data on suicide in the MENA region, particularly in the fast-developing regions of the Arabian Peninsula. In Qatar, there is no registry on the rate of suicide attempts or self-harm, causative factors, methods used or socio-demographic data of cases. These data are crucial in planning services aimed at the reduction in suicidal behavior. Besides, there are no previous clinical or community studies on suicide in Doha, Qatar. This study aimed to estimate the annual incidence of suicide attempt visits to ED in Doha and to investigate trends in suicide in Qatar’s largest Government Hospital, with the objective of producing a nationally recognized body of reliable knowledge. The latter will aid in better understanding and identifying factors that were associated with suicidal attempts. We also wanted to check the documentation of the different risk factors related to suicide to educate clinical staff and policymakers about the need to build a national database of suicide. This would help the country to have a better understanding of patterns of suicide to adopt the most appropriate preventive and intervention plans.

## Methods

This is a retrospective study of all the cases presented to the Emergency Department (ED) of Hamad Medical Corporation (HMC), Doha, Qatar, with self-harm or suicide attempts in the period between July 2011 and June 2012. The study was conducted between March 2013 and January 2014. It was approved by the Institutional review boards of HMC and Weill Cornell Medicine in Qatar (WCM-Q). No written informed consent or information sheet was required as human subjects were not contacted directly and all the data collected were de-identified immediately.

### Setting and Participants

Qatar is a rapidly developing country with a sizeable worldwide expatriate population. Residents in Qatar are made up of 30% Arabs (including Qataris), 60% from the East Asian and Indian subcontinent, and a smaller proportion from Europe, America, and Australasia. The population of Qatar was about 1.70 million people at the time of the study with sex ratio approximately 3.2:1 male to female (Qatar Statistic Authority 2015). HMC is the leading state health care center in Qatar, accepting referrals from primary health care centers and the private sector; the ED is the only state emergency facility in Doha. The Psychiatry Hospital is the only psychiatric facility in the country. It has three male wards (45-bed capacity total) and one female ward (20-bed capacity). All deaths are reported to HMC ED. We further checked the coroner’s office to try to have more information on these cases. Furthermore, at the time of the study, private sector emergency services, of which there were very few, would refer all self-harm cases to HMC ED. Therefore, we believe that the sample assessed was largely representative of the population under study.

The daily patient visits to the ED were about 3,000. All emergency admissions to HMC between July 2011 and June 2012 were reviewed by studying the electronic database. Self-harm and suicidal attempts were identified by employing a thorough, systematic search method, which consisted of filtering cases based on the ICD 10 codes (namely poisoning – accidental or intentional; overdose; gunshot or stabbing; asphyxiation) and utilizing relevant search words (such as suicide, overdose, ingestion, jumping). Cases of self-harm or suicide that were presented to ED and were not admitted to the Psychiatry ward (i.e., discharged, given a referral to the Psychiatry outpatients or admitted to the medical wards) were also scrutinized. All cases admitted to inpatient wards with suicide or self-harm were also extracted. Any inpatient psychiatry admission that mentioned a drug overdose, fire-setting, cutting of self or any body part, stabbing, jumping from a height, jumping in front of traffic, hanging, self-strangulation, alcohol intoxication, ingesting chemicals or foreign objects, burning of self, or banging head against a wall were included. Through employing this systematic search, 616 cases were identified: 143 (26%) were not self-harm or suicide cases, for example, stabbings due to assault or injuries from falls, 165 cases had suicide intentions and were admitted to Psychiatry wards, 105 cases were considered intentional self-harm/suicide but were not admitted to Psychiatry, 150 cases were reported as accidental self-harm (these will be labelled as parasuicide henceforth), and 53 cases had no records available. Thus, the first group that was clearly not self-harm or suicide was excluded from the comparisons. The remaining three groups that we analyzed were: suicide attempt/Admitted (N = 165), reported suicide attempt but not admitted (N = 105), and the parasuicide group with self-harm but reported as accidental (N = 150).

### Measures and Data collection

The research group did an extensive review of the literature and developed questionnaires to cover the various sociodemographic, clinical, and psychiatric features related to suicide. The questionnaires were piloted using 25 records of the patients admitted to psychiatry to assess feasibility. After minor modifications in the questionnaires, a manual with explanations and codes were developed to be used during the study. The research coordinator finalized the questionnaires using the medical records available. The records for the subjects who were only seen in the ED were not thorough, and several measures were missing. Another 30 records were repeated by the same rater within ten days, and excellent intra-rater reliability was established. The nursing staff of the inpatient units had a form to assess specific factors in patients admitted with suicide attempts. These factors covered the presence or absence of the following issues: migration-related concerns, helpless/hopeless themes, psychosis, impulsivity, and sleep problems.

### Statistical analysis

The analyzed data were divided into three groups: suicidal attempt/admitted to Psychiatry (Suicide/Adm), suicidal attempt/not admitted to Psychiatry (Suicide/NAdm), and accidental self-harm with no suicidal intent (parasuicide). Continuous variables (like age) were analyzed as the mean ± standard deviation (SD) while the categorical ones as frequency and percentage. All the variables analyzed were categorized and thus we used the Chi-square test to compare the data between the above groups. We used IBM Statistical Package for Social Sciences (SPSS) for Mac version 24 (IBM Corp, Armonk, NY, 2015) to analyze the data. *P* value was set at 0.05 for level of significance. All analyses were done with Bonferroni corrections for multiple comparisons across subgroups. This type of correction is automated in SPSS and usually based on the significance set level divided by the number of comparisons in each set. Regarding missing data, we calculated the percentage for each variable and category in the study. For the nominal variables, the missing data was added and analyzed as a separate category. When the latter was more than 25%, we did not proceed with the interpretation of the results. Furthermore, if the missing data category was showing significance, then the results were considered invalid.

## Results

### Sample Characteristics by group (Table 1)

**Table 1:** Sociodemographic characteristics by group

Variable	Suicide/Adm N = 165	Suicide/NAdm N = 105	Parasuicide N =150	Total N = 420
Age (years)				
Age ≤ 25	64 (38.8%)	70 (66.7%)^a,c^	69 (46.0%)	203 (48.3%)
Age 26–40	86 (52.1%)^b^	32 (30.5%)	63 (42.0%)	181 (43.1%)
Age ≥ 41	15 (9.1%)	3 (2.9%)	18 (12.0%)^b^	36 (8.6%)
Gender				
Male	82 (49.7%)^b^	25 (23.8%)	82 (54.7%)^b^	189 (45.0%)
Female	83 (50.3%)	80 (76.2%)^a,c^	68 (45.3%)	231 (55.0%)
Marital status				
Single	84 (50.9%)	20 (19.0%)	14 (9.3%)	118 (28.1%)
Married	30 (18.2%)	23 (21.9%)	15 (10.0%)	68 (16.2%)
Divorced/widowed	11 (6.7%)	0 (0.0%)	1 (0.7%)	12 (2.9%)
Separated	40 (24.2%)	0 (0.0%)	0 (0.0%)	40 (9.5%)
Not reported	0 (0.0%)	62 (59.0%)	120 (80.0%)	182 (43.3%)
Children				
Have children	62 (37.6%)	10 (9.5%)	7 (4.7%)	79 (18.8%)
No children	55 (33.3%)	7 (6.7%)	0 (0.0%)	62 (14.8%)
Not reported	48 (29.1%)	88 (83.8%)	143 (95.3%)	279 (66.4%)
Nationality				
East Asia	72 (43.6%)^b^	21 (20.0%)	56 (37.3%)^b^	149 (35.5%)
Far Asia	21 (12.7%)	7 (6.7%)	13 (8.7%)	41 (9.8%)
Qatar	30 (18.2%)	28 (26.7%)	32 (21.3%)	90 (21.4%)
Arabs other than Qatar	37 (22.4%)	45 (42.9%)^a,c^	40 (26.7%)	122 (29.0%)
Western	5 (3.0%)	4 (3.8%)	9 (6.0%)	18 (4.3%)
Educational level				
Up to eighth grade	32 (19.4%)	0 (0.0%)	0 (0.0%)	32 (7.6%)
Above eighth grade	59 (35.8%)	4 (3.8%)	1 (0.7%)	64 (15.2%)
Illiterate	19 (11.5%)	3 (2.9%)	2 (1.3%)	24 (5.7%)
Not reported	55 (33.3%)	98 (93.3%)	147 (98.0%)	300 (71.4%)
Occupation				
Housekeeper	54 (32.7%)	12 (11.4%)	7 (4.7%)	73 (17.4%)
Employed				
Unemployed	32 (19.4%)	6 (5.7%)	6 (4.0%)	44 (10.5%)
Student	40 (24.2%)	0 (0.0%)	2 (1.3%)	42 (10.0%)
Housewife	14 (8.5%)	17 (16.2%)	7 (4.7%)	38 (9.0%)
Not reported	6 (3.6%)	2 (1.9%)	2 (1.3%)	10 (2.4%)
Living situation	19 (11.5%)	68 (64.8%)	126 (84.0%)	213 (50.7%)
Alone	10 (6.1%)	0 (0.0%)	0 (0.0%)	10 (2.4%)
With friends	7 (4.2%)	3 (2.9%)	3 (2.0%)	13 (3.1%)
With family	78 (47.3%)	11 (10.6%)	8 (5.3%)	97 (23.2%)
Employer housing	29 (17.6%)	2 (1.9%)	0 (0.0%)	31 (7.4%)
Work camps	32 (19.4%)	1 (1.0%)	0 (0.0%)	33 (7.9%)
Not reported	9 (5.5%)	87 (83.7%)	139 (92.7%)	235 (56.1%)
Income category				
Low	63 (38.2%)	2 (1.9%)	0 (0.0%)	65 (15.5
Average	23 (13.9%)	0 (0.0%)	1 (0.7%)	24 (5.7%)
Good/supported	9 (5.5%)	0 (0.0%)	0 (0.0%)	9 (2.1%)
Not reported	70 (42.4%)	103 (98.1%)	149 (99.3%)	322 (76.7%)

^a^Higher than the suicide/Adm

^b^Higher than the suicide/NA

^c^Higher than the parasuicide (*p* < 0.05)

The annual incidence rate of suicide attempts visiting ED in Qatar is 159 per 100,000 population where we counted only the cases with suicide intent (165 and 105), and the total population of Qatar of 1.7 million at the time of the study. Chi square tests showed that the following variables were significantly different between the three groups: Age group (χ^2^(4) = 23.73, *p* < 0.001), Gender (χ^2^(2) = 26.18, *p* < 0.001), and Nationality (χ^2^(8) 26.56, *p* = 0.001). Post hoc comparisons showed that more subjects with the age of 26–40 years belonged to the group suicide/Adm when compared to those who were not admitted. The number of subjects with age less than 25 years was significantly higher in the group suicide/NAdm compared to those who were admitted and to those belonging to parasuicide. The latter group had a significantly higher number of subjects 41 years or older compared to those in the suicide/NAdm group. The percentage of males was significantly higher in the suicide/Adm and parasuicide groups compared to those in suicide/NAdm, which had a significantly higher proportion of females. Bonferroni-adjusted post hoc analyses showed that more subjects from East Asia were seen in the groups suicide/Adm and parasuicide than the ones within suicide/NAdm. The proportion of Arabs (other than Qataris) was higher in the group suicide/NAdm compared to the other two groups (Table 1). The other variables (marital status, having children, level of education, occupation, living situation, and income) had a high percentage of missing data, and thus further statistical analysis was not appropriate.

During the period of our study 37 cases were identified as death by suicide from the hospital records: all males in their mid 30 s, one from Qatar, two Arabs, and the rest are expatriates. They did not have any past medical or psychiatric records. The majority (95%) used hanging with a rope as the method of suicide. No other data are available on these cases.

### Clinical Characteristics of the Sample (Table 2)

**Table 2:** Clinical characteristics by group (retrospective)

VariableVariable	Suicide/Adm N = 165	Suicide/NAdm N = 105	Parasuicide N = 150	Totals N = 420
Psychiatry consulted				
No	–	15 (14.3%)	110 (73.3%)^a^	125 (49.0%)
Yes	–	83 (79.0%)^b^	38 (25.3%)	121 (47.5%)
Left before	–	7 (6.7%)^b^	2 (1.3%)	9 (3.5%)
Psychiatric diagnosis				
None	32 (19.4%)	29 (27.6%)	9 (6.0%)	70 (16.7%)
Childhood-onset disorders	65 (39.4%)	1 (1.0%)	1 (0.7%)	67 (16.0%)
Mood disorders	45 (27.3%)	17 (16.2%)	2 (1.3%)	64 (15.2%)
Psychotic disorders	13 (7.9%)	2 (1.9%)	0 (0.0%)	15 (3.6%)
Substance use disorders	1 (0.6%)	0 (0.0%)	0 (0.0%)	1 (0.2%)
Personality disorders	9 (5.5%)	3 (2.9%)	0 (0.0%)	12 (2.9%)
Not reported	0 (0.0%)	53 (50.5%)	138 (92.0%)	191 (45.5%)
Past psychiatric diagnosis				
None	115 (69.7%)	38 (36.2%)	20 (13.3%)	173 (41.2%)
Mood disorders	12 (7.3%)	2 (1.9%)	6 (4.0%)	20 (4.8%)
Psychotic disorders	6 (3.6%)	3 (2.9%)	1 (0.7%)	10 (2.4%)
Substance use disorders	1 (0.6%)	0 (0.0%)	0 (0.0%)	1 (0.2%)
Personality disorders	9 (5.4%)	1 (1.0%)	0 (0.0%)	10 (2.3%)
Not reported	22 (13.3%)	61 (58.1%)	123 (82.0%)	206 (49.0%)
Family psychiatric disorder				
Yes	6 (3.6%)	2 (1.9%)	0 (0.0%)	8 (1.9%)
No	130 (78.8%)	17 (16.2%)	17 (11.3%)	164 (39.0%)
Not reported	29 (17.6%)	86 (81.9%)	133 (88.7%)	248 (59.0%)
Substance use				
None	106 (64.2%)	18 (17.1%)	14 (9.3%)	138 (32.9%)
Nicotine	21 (12.7%)	1 (1.0%)	0 (0.0%)	22 (5.2%)
Alcohol	4 (2.4%)	0 (0.0%)	2 (1.3%)	6 (1.4%)
Marijuana	1 (0.6%)	0 (0.0%)	0 (0.0%)	1 (0.2%)
Nicotine and alcohol	6 (3.6%)	0 (0.0%)	0 (0.0%)	6 (1.4%)
Nicotine and marijuana	5 (3.0%)	0 (0.0%)	0 (0.0%)	5 (1.2%)
Not reported	22 (13.3%)	86 (81.9%)	134 (89.3%)	242 (57.6%)
Medical history/treatment				
Present	26 (15.8%)	10 (9.5%)	20 (13.3%)	56 (13.3%)
Absent	117 (70.9%)	23 (21.9%)	14 (9.3%)	154 (36.7%)
Not reported	22 (13.3%)	72 (68.6%)	116 (77.3%)	210 (50.0%)
Disposition from ED				
Discharged home	0 (0.0%)	42 (40.0%)	129 (86.0%)	171 (67.1%)
Discharged AMA	0 (0.0%)	15 (14.3%)	5 (3.3%)	20 (7.8%)
Admitted to medicine	0 (0.0%)	7 (6.7%)	7 (4.7%)	14 (5.5%)
Admitted to psychiatry	165 (100.0%)	0 (0.0%)	0 (0.0%)	165 (100.0%)
Outpatient psychiatry	0 (0.0%)	37 (35.2%)	9 (6.0%)	44 (18.1%)
Absconded	0 (0.0%)	4 (3.8%)	0 (0.0%)	4 (1.6%)

^a^Higher than the suicide/NA

^b^Higher than the parasuicide (*p* < 0.05)

Psychiatry saw all the subjects in the suicide/Adm group before being admitted. Psychiatry services were consulted on the majority of the subjects in the suicide/NAdm group (79.0%) but not on 73.3% of the parasuicide group (χ^2^(2) 86.47, *p* < 0.001). Nine cases of the whole sample left before being seen by Psychiatry, and four subjects absconded (Table 2). Only two subjects were identified as repeat suicidal attempt during the period of the study.

In the group of suicide/Adm, the majority did not have past (69.7%) or family (78.8%) psychiatric history, but after admission the most common diagnoses were childhood-onset (39.4%; intellectual disabilities, attention deficit hyperactivity disorder, autism spectrum disorder, and tic disorder) and mood (27.3%) disorders. Only 22.6% reported a history of substance use (mainly nicotine followed by alcohol and marijuana) and 15.8% had a history of chronic medical disorder. The other two groups had significant missing data on the clinical measures, and thus further statistical analysis was not pursued. Concerning the cases that were not admitted to Psychiatry, 67% were discharged home, 5.5% admitted to Medicine, and 18.1% were referred to outpatient Psychiatry (Table 2).

### Factors relevant to suicide assessment (Table 3)

**Table 3:** Factors relevant to suicide risk by group (retrospective)

Variable	Suicide/Adm N = 165	Suicide/NAdm N = 105	Parasuicide N = 150	Total N = 420
Method of attempt				
Overdose	82 (78.1%)^b,c^	78 (52.0%)	69 (41.8%)	229 (54.5%)
Ingest chemical/object	13 (12.4%)	67 (44.7%)^a,c^	19 (11.5%)	99 (23.6%)
Cut/stab/burning self	6 (5.7%)	4 (2.7%)	46 (27.9%)^a,b^	56 (13.3%)
Hanging with robe	2 (1.9%)	1 (0.7%)	21 (12.7%)^a,b^	24 (5.7%)
Jumping from heights	2 (1.9%)	0 (0.0%)	10 (6.1%)	12 (2.9%)
Previous suicidal attempts				
Present	3 (2.9%)	0 (0.0%)	30 (18.2%)	33 (7.9%)
Absent	37 (35.2%)	21 (14.0%)	115 (69.7%)	173 (41.2%)
Not reported	65 (61.9%)	129 (86.0%)	20 (12.1%)	214 (51.0%)
Family history of suicide				
Yes	1 (1.0%)	0 (0.0%)	4 (2.4%)	5 (1.2%)
No	17 (16.2%)	15 (10.0%)	126 (76.4%)	158 (37.6%)
Not reported	87 (82.9%)	135 (90.0%)	35 (21.2%)	257 (61.2%)
Psychosocial stress				
Marital/family	55 (52.4%)	3 (2.0%)	62 (37.6%)	120 (28.6%)
Friends/colleagues	10 (9.5%)	1 (0.7%)	15 (9.1%)	26 (6.2%)
Financial	2 (1.9%)	0 (0.0%)	10 (6.1%)	12 (2.9%)
Work	18 (17.1%)	2 (1.3%)	9 (5.5%)	29 (6.9%)
Not reported	20 (19.0%)	144 (96.0%)	69 (41.8%)	233 (55.5%)

^a^Higher than the suicide/Adm

^b^Higher than the suicide/NA

^c^Higher than the parasuicide (*p* < 0.05)

The most common method of self-harm was an overdose on medications (54.5%) followed by ingesting chemicals or objects (23.6%), self-cut or burn (13.3%), rope hanging (5.7%), and then jumping from heights (2.9%) (see total in Table 3). The method of self-harm was significantly different between the three groups (χ^2^(8) 137.49, *p* < 0.001). Post hoc comparisons showed that the number of subjects with overdoses was significantly higher in the suicide/Adm than the ones in the other two groups, while the group suicide/NAdm showed higher number with ingesting chemicals/objects than the other two groups. The number of subjects with self-cut/burn or hanging with rope was significantly higher in the parasuicide group when compared to the other two groups (Table 3). In the group admitted to psychiatry, the nurse assessment showed that 60.6% had hopeless/hopeless themes, 40.4% impulsivity, 35.2% sleep problems, and 19.4% psychotic symptoms.

## Discussion

The main aim of this study was to assess the annual incidence of the suicide attempts visiting ED and the various factors associated with suicide attempts and self-harm behaviors in Doha, Qatar. In this section we will discuss first the committed suicide cases then cover the various sociodemographic and clinical variables related to suicide attempts and self-harm.

### Completed suicide group

Our data on the cases with fatal suicide (N = 37) were limited where all were males, mid 30 s, and the majority were expatriates who died by hanging. Studies from the Arabic Peninsula showed similar reports in regard to fatal suicide. The profile of suicide cases in UAE showed that they were mostly males, single, employed, older than 30 and expatriates. The suicide rate was seven times higher in expatriates compared to nationals (Dervic et al. 2012). Review of the police records in Kuwait (Al-Waheeb and Al-Kandary 2015) showed that the most common method of suicide was hanging (60%) and the majority were expatriates (87%). A similar pattern was also reported in Kingdom of Saudi Arabia (KSA) (Elfawal 1999; Helaly et al. 2015), Bahrain (Al Ansari et al. 2007) and UAE (Dervic et al. 2012; Koronfel 2002). A review of the Arab studies using police and government records (Karam et al. 2008) reported that the annual rate of completed suicide ranged from 1.1 to 6.2 per 100,000 population where they were mostly males, 20–40 years old with hanging as the most common method in UAE and KSA while in Jordan and Lebanon the methods used were mostly gunshots in males and self-immolation or pesticide poisoning in females. In the Arabic Peninsula, the majority of the population are expatriates, which could explain why the majority of the suicide cases were expatriates; for example, in Qatar over 70% of the population are non-Arabs (Qatar Statistic Authority 2015). A recent study from Iraq (Abbas et al. 2018) showed that the majority of suicide cases were less than 30 years old and the most common methods were hanging (41%), gunshots (31.4%), and self-immolation (19.2%). Only 24.1% were known to have psychiatric disorder where depression was the predominant one. Only a small percentage had psychological trauma (15.5%) or financial problems (12.4%). The lack of any cases using guns in Qatar compared to the high percentage in Iraq is probably related to availability where guns were more readily available in Iraq that suffered many internal conflicts in the last decade. Similarly, A systematic review from studies in EMR reported that the most common methods were hanging (39.7%), self-immolation (17.4%), and poisoning (20.3%) (Morovatdar et al. 2013). It has also been noted that the rates of suicide appear lower amongst Muslim countries, regardless of whether the population belongs to several religious groups (Lester 2006). Nevertheless, suicide is considered “haram”, or a forbidden act in Islam, thus resulting in its inevitable underreporting (Sarfraz and Castle 2002). Pritchard and Amanullah (2007) argued that inconsistencies in suicide rates in the Middle East might be a repository for hiding culturally unacceptable suicides. However, compared to other countries, suicide rates are lowest in the EMR (Malakouti et al. 2009). Several explanations were given to this observation: many of the EMR countries do not have a suicide or self-harm registry system, which would result in under-reporting, religion plays a significant protective factor from suicide, the stigma associated with self-harm is significantly high, and the cause of death in some cases may not be reported as suicide for a variety of social reasons (Sarfraz and Castle 2002; Malakouti et al. 2015).

### Profile of the Three Self-harm Groups

The annual incidence of suicide attempts visiting ED was 159 per 100,000 population of Qatar. When we looked at these rates in the Qataris and Arabs (30% of the total population) only, this rate was 27 per 100,000 Qatari/Arab population in Qatar. The majority of serious suicide attempters who needed inpatient psychiatric treatment were males, from East Asia, and with ages between 26 and 40 years (Table 1). In the group Suicide/NAdm we had more females who were younger (< 25 years) compared to the other two groups. In this group, most of them had no past psychiatric history, but the common discharge diagnoses were childhood-onset and mood disorders. Overdose on medications was the most common method in those who had suicide attempts and also in those with self-harm without suicide intent. In patients admitted after suicide attempts, many of them had stress related to marital/family issues (Table 3). The frequencies of the risk factors assessed in the inpatient group by nurses were: helpless/hopeless themes (60.6%), impulsivity (40.6%), sleep problems (35.2%), and psychosis (19.4%) (Table 4). There were significant missing data in the other demographic variables and thus proper analysis was not feasible. In the three self-harm groups, the majority were females (55%). These suicidal rates and the profile on age and gender are similar to the ones reported for the ED visits in US (Canner et al. 2018), India (Saddichha et al. 2010), Japan (Kawashima et al. 2014) but lower than those from Greece (Fountoulakis et al. 2015). As in our results, a review of the community based studies from the Arab countries (Karam et al. 2007) showed that the annual rates of suicide attempts ranged from 1.9 to 127 per 100,000 population. The majority were females, 15–25 years old, single, unemployed, with overdose being the most common method and depression as the most common psychiatric disorder. The higher prevalence of suicide attempts in females was also demonstrated in a review on Middle Eastern Muslim females (Rezaeian 2010). The psychosocial profile of cases presenting with drug overdose in KSA reported that they were also mostly young (18–26 years old) Saudi females (Al-Jahdali et al. 2004; Bakhaidar et al. 2015).

**Table 4:** Suicide risk factors in the group admitted to psychiatry wards (N = 165)

Variables	N (%)
Migration issues	
Wish to travel home	11 (6.7%)
Unable to travel home	9 (5.5%)
Migrated in < 6 months	25 (15.2%)
Not reported	120 (72.7%)
Helpless/hopeless themes	
Absent	65 (39.4%)
Present*	100 (60.6%)
Psychosis	
Absent*	133 (80.6%)
Present	32 (19.4%)
Impulsivity	
Absent*	98 (59.4%)
Present	67 (40.6%)
Sleep problems	
Absent*	107 (64.8%)
Present	58 (35.2%)

*Significantly more than the other category (*p* < 0.05)

In regard to ethnicity, the common nationality in the groups Suicidal/Adm and Parasuicide was East Asia, while in the group Suicide/NAdm it was Arabs (non-Qatari). Looking at the percentages of the different nationalities in the three groups, we can see that Qataris and Arabs (40–70%) are over-represented compared to the ethnic distribution in Qatar (Table 3). Other multiethnic countries also reported over-representation of certain nationalities in the cases seen in ED for suicidal behaviors like Singapore where Indians were over-represented (Choo et al. 2017). A systemic review from United Kingdom (UK) (Al-Sharifi et al. 2015) also reported that self-harm is more common in Black females and that religion is a modifying factor when assessing the suicide risk in the different ethnicities and cities in UK. We could not find other studies or registries from the Arab countries that assessed self-harm cases visiting ED. Thus, it seems that the rates of suicidal attempts among Arabs and Muslims are not significantly lower than other countries or non-Muslims. This was also noted in review studies from EMR where the authors (Lester 2006) argued that the under-reporting of suicide may be in part due to its illegality or the high stigma attached to self-harm.

Another universal observation that is worth mentioning is the fact that the majority of the parasuicide cases were discharged home, and from those with suicide attempt/not admitted, only about 35% of the cases were referred to outpatient psychiatry clinics (Table 2). A recent study from Tochigi, Japan reported also that only half of the patients seen in ED after suicidal attempts, were referred to Psychiatry (Chihara et al. 2018). This issue needs further attention as such cases need closer following because of the increased risk of further suicidal attempts that can become lethal (Haw et al. 2003; Runeson et al. 2010).

## Limitations

This study has several strengths in regard to study design and being the only study from Qatar but it has also limitations that are worth discussing: First, it is a retrospective study and thus many factors could not be controlled properly. Second, many demographic and psychiatric variables are missing and thus a well-planned prospective study is needed to cover these aspects. Third, a longer period of assessment is better to have a larger sample to estimate the annual suicide rates and generalize the significant findings. Fourth, in a rapidly growing country like Qatar, more regular and updated assessments are needed for better planning of the intervention and prevention procedures.

## Conclusions

Our results showed that more expatriate single males are at risk to commit suicide in Qatar and more screening of their biopsychosocial profiles is warranted in any future plans to prevent these suicides. The Arabs are more at risk to attempt suicide and further measures to minimize access to medications and poisons are needed together with better psychiatric screening and following of self-harm cases when they are in contact with healthcare facilities. Our data should be used effectively to have better documentation of all self-harm cases in Qatar, allowing more reliable registries to scrutinize the proper assessment of all self-harm cases and the necessary interventions for treatment and adequate follow-up. The results could also guide policies to plan for suicide prevention and early interventions in Qatar.
